# *Nocardia* genomes are a large reservoir of diverse gene content, biosynthetic gene clusters, and species-specific genes

**DOI:** 10.1128/mbio.00947-25

**Published:** 2025-05-23

**Authors:** Kiran Kumar Eripogu, Chun-Ping Yu, An-I Tsai, Jinn-Jy Lin, Hsiao-Ching Lin, Wen-Hsiung Li

**Affiliations:** 1 Biodiversity Program, Taiwan International Graduate Program, Academia Sinica, Taipei, Taiwan; 2 Biodiversity Research Center, Academia Sinica (BRCAS) 38017 , Taipei City, Taiwan; 3 Department of Life Sciences, National Taiwan Normal University (NTNU) 34879, Taipei City, Taiwan; 4 National Center for High-performance Computing, National Applied Research Laboratories, Hsinchu, Taiwan; 5 Institute of Biological Chemistry, Academia Sinica 38017, Taipei City, Taiwan; 6 Department of Ecology and Evolution, University of Chicago, Chicago, Illinois, USA; Emory University School of Medicine, Atlanta, Georgia, USA

**Keywords:** *Nocardia*, genome mining, open pangenome, biosynthetic pathways

## Abstract

The *Nocardia* genus represents a largely untapped source of valuable secondary metabolites, yet its biosynthetic potential, gene content, and evolutionary history remain underexplored. By analyzing 263 genomes across 88 species, we found that *Nocardia* varies greatly in genome size and gene content. It exhibits an open pangenome with a small core genome (<900 genes) and high genomic fluidity (0.76), indicating high gene turnover. A large proportion (75%) of its genes are species-specific, indicating high genomic plasticity. Average nucleotide identity (ANI) analysis confirmed taxonomic relationships, with most species showing ANI values (80–85%). *N. globerula* showed an ANI of ~84% with *Rhodococcus erythropolis*, supporting its reclassification under *Rhodococcus*. The biosynthetic capabilities of the *Nocardia* genus are striking, with the presence of >8,000 biosynthetic gene clusters (BGCs), dominated by type 1 polyketide synthase, terpenes, and non-ribosomal polypeptide synthetases, establishing *Nocardia* as the Actinomycetota genus that has the largest biosynthetic repertoire. Around 35% of BGCs remain uncharacterized, suggesting *Nocardia*’s high potential for novel natural product discoveries. Our study is the first to identify a prodigiosin BGC in *Nocardia*. Network analysis revealed complex evolutionary connections between *Nocardia*’s gene cluster families (GCFs) and MIBiG reference BGCs, suggesting evolutionary changes, including gene gains and losses, that may have influenced the genus’s BGC diversity and composition. Synteny analysis uncovered conserved and unique gene arrangements across *Nocardia* and related genera, mostly with core genes conserved in Actinomycetota. Our study addressed unmet clinical and biotechnological challenges while revealing evolutionary mechanisms that shape microbial diversity and adaptability.

**IMPORTANCE:**

Understanding the genomic diversity and biosynthetic potential of microorganisms is instrumental for addressing issues in microbial evolution, natural product discovery, and host-microbe interactions. *Nocardia*, a bacterial genus known for its opportunistic pathogenicity, represents an underexplored group of immense genomic diversity and biosynthetic capabilities. This study employed genome mining to reveal the open pangenome of *Nocardia* and identified an extensive repertoire of BGCs, including novel clusters with the potential to produce therapeutically significant compounds such as prodigiosin-related compounds. By integrating genome mining, phylogenetics, and synteny analysis, this study provides insights into how genomic plasticity, species-specific genes, and evolutionary changes such as gene gains and losses that contribute to *Nocardia*'s biosynthetic diversity and evolution. These findings contribute to advancing microbial genomics, evolution, and biotechnology by uncovering the potential of *Nocardia* to address challenges in infectious diseases and natural product discovery. This study exemplifies how genome mining can illuminate the ecological and clinical significance of microbial diversity.

## INTRODUCTION

Exploration of bacterial genomes can reveal untapped resources for bioactive compounds with potential clinical and biotechnological applications (1–3). Indeed, mining bacterial genomes has uncovered many new bioactive compounds by identifying biosynthetic gene clusters (BGCs) in the genomes (4–6). These BGCs are responsible for the synthesis of diverse secondary metabolites including antibiotics, immunosuppressants, and antimicrobials, which are vital for combating infectious diseases. Genome mining tools like antiSMASH and databases such as MIBiG have enabled the prediction and cataloging of both known and novel BGCs (5, 7). Pathogenic bacterial genera are particularly intriguing as sources of secondary metabolites due to their adaptation to diverse ecological niches or host environments. The *Streptomyces* (8), *Pseudoalteromonas* (9)*,* and *Nocardia* (10) genera have emerged as high producers of secondary metabolites. These secondary metabolites were found to possess wide clinical applications, contributing to the development of bioactive compounds used to combat pathogenic infections (11–13). Their biosynthetic potential highlights the interplay between pathogenicity and natural product biosynthesis.

*Nocardia* is known for producing over 50 bioactive compounds while encompassing opportunistic pathogens responsible for causing diseases like nocardiosis, affecting the cutaneous, pulmonary, and nervous systems (14–16). Despite its pathogenicity, *Nocardia* species harbor diverse BGCs that contribute to the synthesis of a wide range of bioactive compounds, including nocardicin A, brasilicardin A, nargenicin, and nocobactin A (12, 17). These compounds belong to various biosynthetic classes such as non-ribosomal polypeptides, polyketides, thiopeptides, and terpenes, highlighting *Nocardia*’s pharmaceutical potential. However, the complex taxonomy of the genus *Nocardia* poses challenges for species-level identification, creating challenges for accurate clinical diagnoses and emphasizing the need for more precise classification methods (14–16). The genus’s limited genomic representation in literature and databases has further hindered efforts to understand its biosynthetic capabilities. Recent advancements in genome mining (10) shed light on the diversity and distribution of its gene clusters, reaffirming *Nocardia*’s status as a promising yet underexplored genus for natural product discovery. Beyond its biosynthetic potential, understanding the genomic and ecological diversity of *Nocardia* will provide insights into microbial adaptation, community interactions, and their broader implications for ecosystem health and evolutionary biology.

Building on the above issues and insights, the objective of this study was to comprehensively explore the genomic and biosynthetic diversity of *Nocardia*. Specifically, we aimed to (i) expand the genomic data set by incorporating a broader range of *Nocardia* assemblies and annotations; (ii) perform a pangenome analysis to assess genomic plasticity, including gene content variation among species; and (iii) investigate the extent and the evolutionary trajectories of BGCs for a better understanding of the mechanisms underlying secondary metabolite production. Together, these objectives provide a framework to advance our understanding of *Nocardia*’s role in natural product discovery and its broader implications for evolutionary biology and therapeutic applications.

## RESULTS AND DISCUSSION

**Fig 1:**
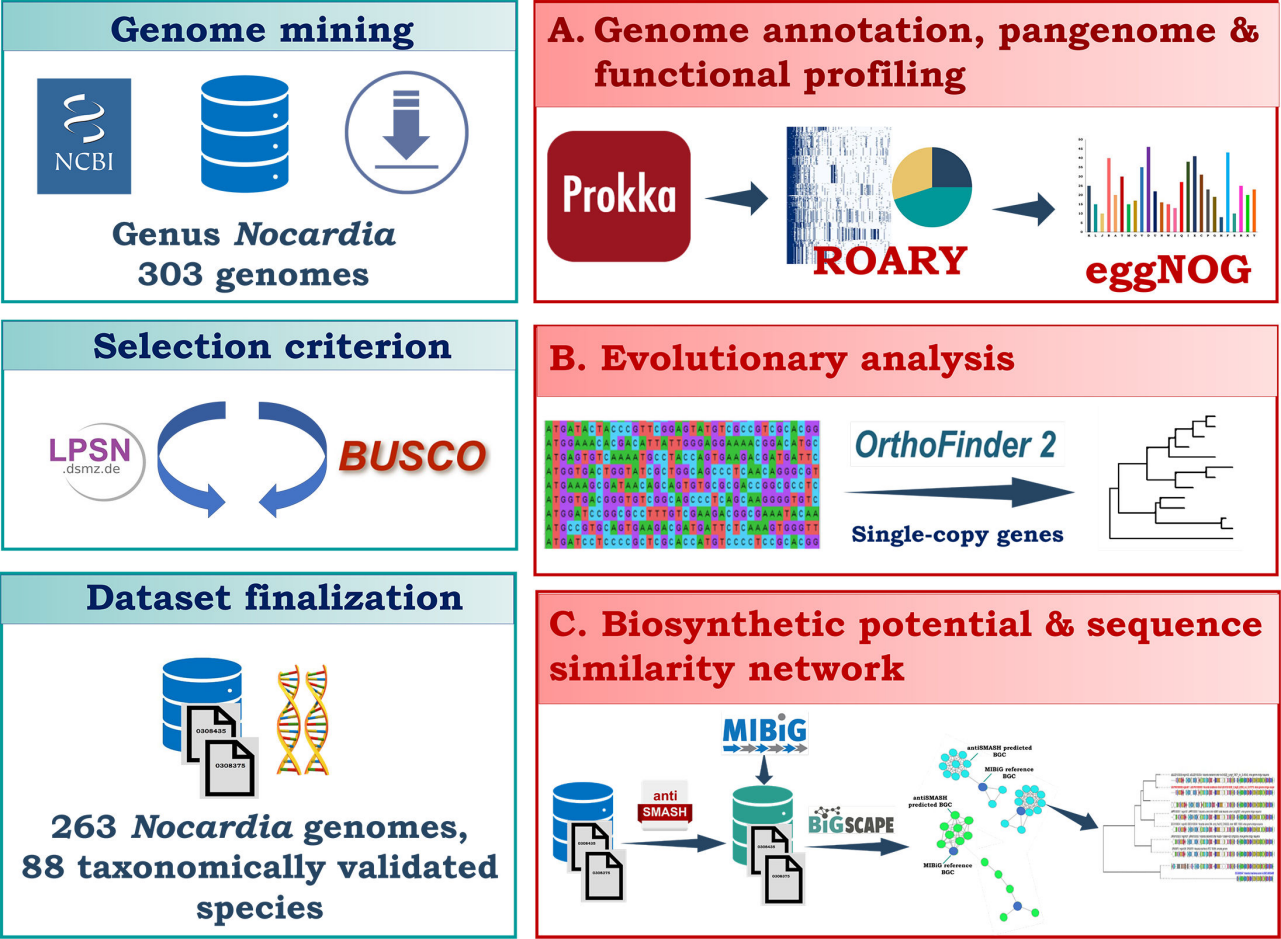
A schematic representation of the integrated approach of genome mining, pangenome analysis, phylogenetic reconstruction, evolutionary analysis, and biosynthetic gene cluster identification.

We collected 303 *Nocardia* genomes from the NCBI GenBank, and following the selection criteria in the LPSN database (https://lpsn.dsmz.de/genus/Nocardia), we identified 263 genomes representing 88 taxonomically validated *Nocardia* species. The selected genomes underwent gene reannotation, pangenome analysis, BGC identification, functional profiling, phylogenomic analysis, and investigation of BGC evolution (see Materials and Methods) (Fig. 1). This integrated methodology provided a comprehensive examination of both genomic and functional diversity among the *Nocardia* species studied.

### High-quality genomes reveal diversity and functional variability

We assessed the genome assembly quality using BUSCO, and it revealed that our genome assemblies were of high quality, with an average BUSCO completeness score as high as ~98%. The *Nocardia* genomes studied had an average size of 7.64 Mbp (range: 5–10 Mbp) and an average G + C content of 68.5% (range: 6–72%) (Table S1). Each *Nocardia* genome was reannotated using Prokka, and it revealed an average of 6,880 genes/genome, with significant variation across strains and species, ranging from 4,682 genes (*N. jinanensis* NBRC 108249) to 9,695 genes (*N. jiangxiensis* NBRC 101359). On average, >50% of the annotated genes are hypothetical proteins, consistent with other studies of *Nocardia* species (18, 19), highlighting challenges in functional annotation and the potential of discovering novel biosynthetic capabilities (Table S2; Fig. S1). Strain-specific variability observed within the same species such as *N. abscessus*, *N. cyriacigeorgica, N. farcinica,* and *N. terpenica* suggests that each *Nocardia* strain had a distinct genetic makeup and may possess unique genetic elements. Our findings align with those of previous studies (18–21). However, gaps such as incomplete assemblies and environmental underrepresentation indicate the need for broader sampling. Currently, ~70% of the genomes deposited at NCBI GenBank were isolated from humans, reflecting the clinical relevance of these bacteria in causing infections like nocardiosis, but limiting our understanding of *Nocardia*’s diversity in non-clinical environments.

### Pathogenicity classification of *Nocardia* species

Our classification of 88 *Nocardia species* leveraging the richness of literature and public databases revealed that 84 species (95.5%) are pathogenic, and the remaining four are non-pathogenic (i.e., with no documented pathogenicity) species (4.5%) (*N. yunnanensis*, *N. stercoris*, *N. aurea*, and *N. bovistercoris*). Pathogenic species were classified into two groups (see Materials and Methods). Risk Group 1 (51 species, 61%) are species that primarily affect immunocompromised individuals or are linked to non-human hosts such as fish (*N. salmonicida*), oysters (*N. crassostreae*), and blueberries (*N. vaccinii*). Risk Group 2 species are capable of causing severe infections in both humans and animals, such as nocardiosis. It includes major zoonotic pathogens, like *N. africana*, *N. asteroides*, and *N. brasiliensis* (Table S3).

Our study improves upon the previous study (22), which classified 53 species into a binary model (established/putative) based solely on human infections. We expand this by introducing a risk-based classification that differentiates species by infection severity and host range, providing a broader and more clinically relevant framework. Our analysis incorporates pathogenicity in vertebrates, invertebrates, and plants, offering a holistic ecological and epidemiological perspective. Additionally, we refine pathogenicity assessment by integrating LPSN, GLoBI, BacDive, and peer-reviewed literature, ensuring a more data-driven and cross-validated classification. Our classification also differentiates species based on host specificity and infection patterns, and these annotations are also reflected in Table S3. However, our classification remains limited by existing clinical reports. Many species remain underexplored due to sampling biases toward human infections, rather than their complete ecological spectrum.

### Average nucleotide identity (ANI) analysis

The ANI analysis, performed using FastANI v1.34 (23) and visualized through ANIclustermap v1.4.0 (24), revealed clear species- and strain-level genomic similarity patterns among *Nocardia* species (see Materials and Methods). Most *Nocardia* species exhibited ANI values between 80% and 85%, indicating species-level boundaries and confirming their taxonomic classification. Within this range, species such as *N. beijingensis* and *N. araoensis* displayed ANI values around 81%–83%, suggesting a closer evolutionary relationship. At the strain level, high ANI values (>95%) were observed among strains of *N. farcinica*, *N. seriolae*, *N. cyriacigeorgica*, *N. terpenica*, *N. brasiliensis*, and *N. abscessus*, confirming their classification within the same species. These results highlight the genomic diversity within *Nocardia*, with most species showing ANI values consistent with their current taxonomic classifications, while lower ANI values among certain species suggest potentially distant taxonomic groups. Notably, species like *N. gamkensis* and *N. jinanensis* showed ANI values below 80%, indicating substantial genetic divergence from other *Nocardia* species. This level of divergence suggests the presence of unique evolutionary lineages that may warrant further taxonomic reevaluation.

The FastANI results provide genome-wide evidence for the misclassification of *N. globerula*. While most *Nocardia* species exhibited ANI values between 80% and 85%, *N. globerula* showed no significant alignment (NA) with all *Nocardia* species, indicating that it shares too little genomic similarity to compute ANI (Fig. S2). This strongly suggests that *N. globerula* is genetically distinct from the *Nocardia* genus. In contrast, *N. globerula* exhibited 83.5% ANI with *Rhodococcus erythropolis*, indicating a closer genomic relationship to *Rhodococcus* than to any *Nocardia* species. Given that ANI values below 80% are indicative of distinct genera, the ANI results strongly suggest that *N. globerula* represents a misclassification within the *Nocardia* genus.

### Phylogenomic analysis

To further explore the evolutionary relationships among these classified species, a phylogenetic tree of 88 high-quality *Nocardia* genomes was constructed, using the concatenated protein sequences of 110 single-copy orthologous genes (see Materials and Methods) (Table S4). These genes were chosen based on their presence as single-copy orthologs across all species studied, a widely used strategy in bacterial phylogenomics (25–27), as it ensures a consistent phylogenetic signal and minimizes distortions from gene duplications or genetic recombination. This approach enhances the reliability and accuracy of phylogenetic inference and provides a stable framework for understanding *Nocardia* evolution.

**Fig 2:**
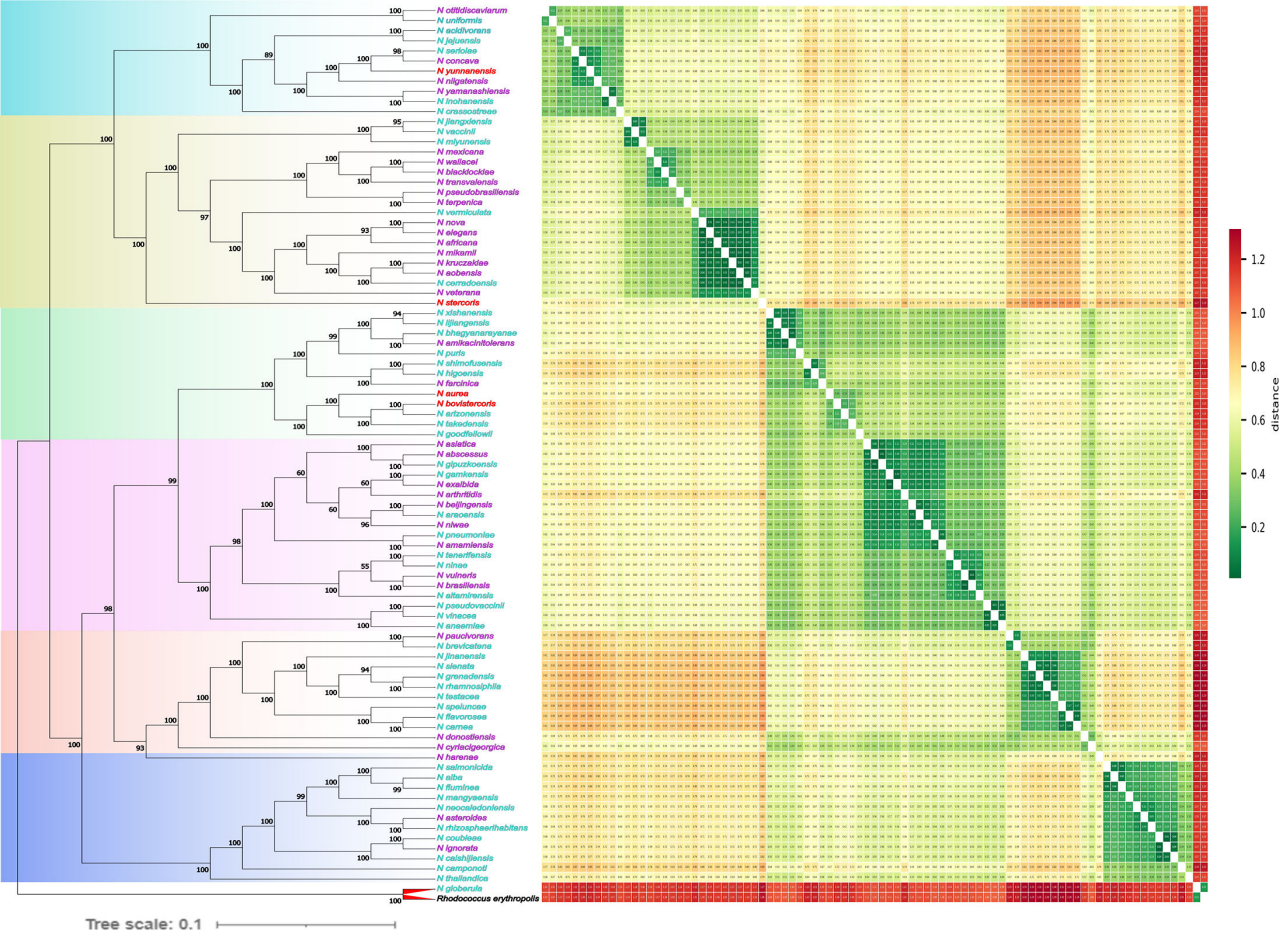
Maximum likelihood (ML) tree of the 88 *Nocardia* species under study. The phylogenetic tree is based on the concatenated 110 single-copy genes with *Rhodococcus erythropolis* NBRC 15567 as an outgroup (left panel). Bootstrap values, calculated from 1,000 replicates, are displayed on the nodes. The phylogenetic tree is color-coded to highlight the six major phylogroups (clades). Additionally, to enhance visualization, species are color-coded for non-pathogenic species (red), risk group 1 pathogens (purple), and risk group 2 pathogens (turquoise blue). The heatmap illustrates the patristic distance matrix among the 88 *Nocardia* species (right panel).

The tree consists of six major phylogroups (clades) supported by high bootstrap values. Differences in branch lengths among species indicate variations in genetic distances between species, which may reflect sequence variations and phylogenetic clustering patterns (Fig. 2). Within Clade I, *N. otitidiscaviarum* and *N. uniformis* have short branch lengths and a patristic distance of only 0.21 (measured as the number of amino acid substitutions per residue site), suggesting a close evolutionary relationship. In contrast, some species in Clade IV, such as *N. stercoris* and *N. harenae,* have relatively longer branch lengths and a larger patristic distance of 0.88, indicative of greater genetic differentiation. The hierarchical structure of the tree is mirrored by the patristic distances, which reflect the genetic relatedness and clustering patterns within *Nocardia*.

*Rhodococcus erythropolis* NRBC 15567 was used as the outgroup in our phylogenetic analysis. This species was chosen due to its taxonomic placement within Corynebacteriales, being closely related to *Nocardia,* while remaining taxonomically distinct, making it an appropriate reference for rooting the phylogenetic tree. This placement highlights the evolutionary separation between the two genera, *Nocardia* and *Rhodococcus*. In Fig. 2, *N. globerula* clusters closely with *Rhodococcus erythropolis,* with a bootstrap value of 100. This high genetic similarity between *N. globerula* and *Rhodococcus erythropolis* is consistent with that in prior studies (28–30). The combined evidence from genome-wide ANI (see above) and phylogenomic analysis suggests that it would be appropriate to classify *N. globerula* under *Rhodococcus* rather than *Nocardia*. Based on our findings and literature support, we recommend the reclassification of *N. globerula* into *Rhodococcus*.

Beyond the taxonomic implications of *N. globerula*, the phylogenetic tree also provides insights into the distribution of *Nocardia* species across clades, further revealing their evolutionary dynamics and pathogenic potential. Clade II, comprising ~65% of species, such as *N. nova*, *N. terpenica,* and *N. africana,* is highly pathogenic, causing severe infections in humans, likely due to specific virulence genes that enhance their ability to infect hosts. In contrast, Clades IV and V exhibit variable virulence, including low-risk and high-risk pathogenic species. Some non-pathogenic species may likely have lost their pathogenicity over time. While some clades show a higher proportion of pathogenic species, non-pathogenic species are interspersed throughout the tree. This suggests that pathogenicity is not strictly clade-dependent but may have evolved or lost through independent events across different lineages. Instead, certain lineages may have acquired virulence traits that confer a selective advantage in pathogenic interactions. These traits likely emerged through a combination of gene acquisition, gene loss, and genomic arrangement, as seen in other bacterial species (31, 32). While horizontal gene transfer (HGT) remains a possibility, no direct analysis was conducted to confirm HGT events. Further studies are needed to determine the precise evolutionary mechanisms driving these differences. This also raises questions about the genetic and ecological factors influencing pathogenic potential within specific phylogenetic lineages.

### Open pangenome and genomic plasticity

**Fig 3:**
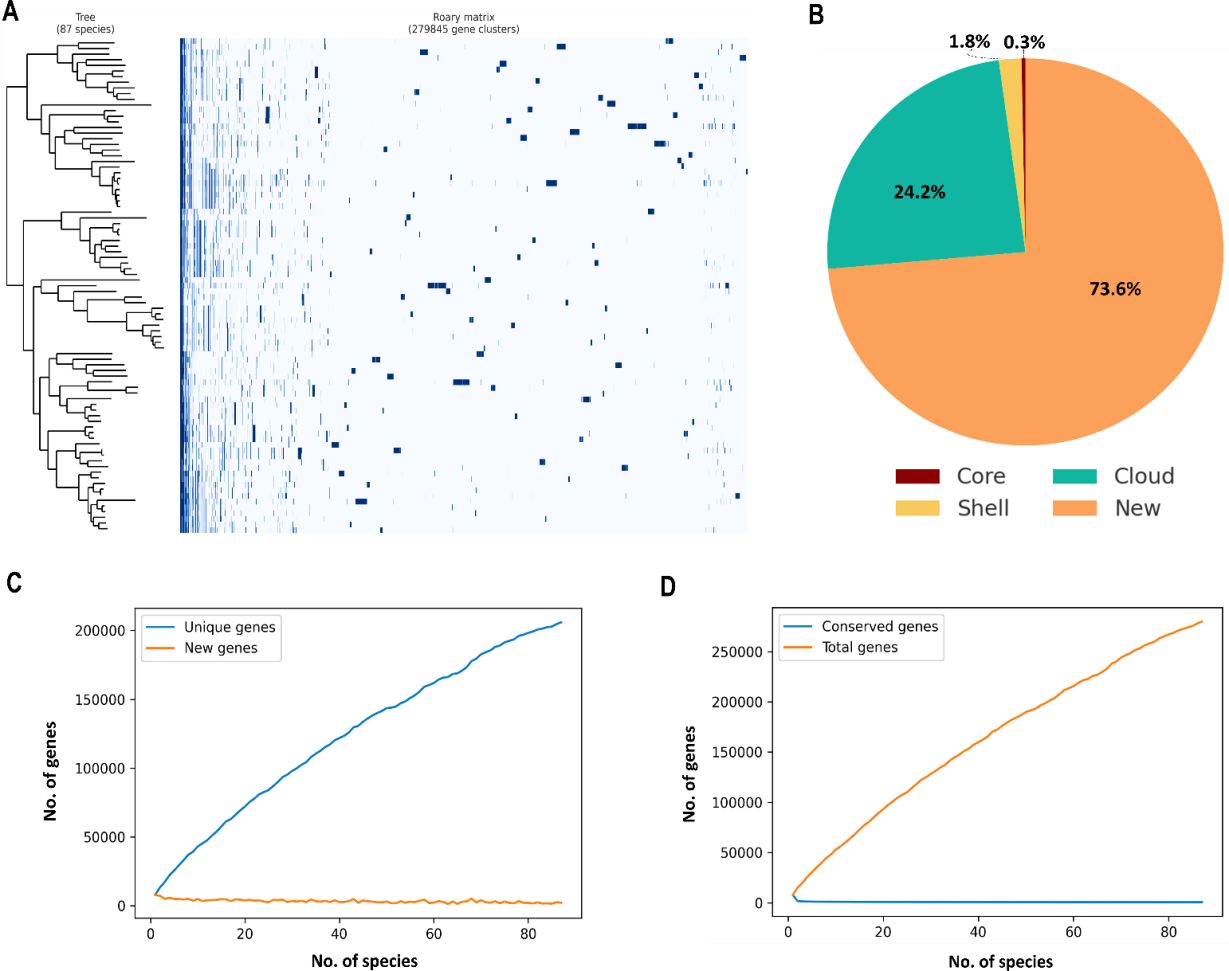
Pangenome analysis of 87 species of the genus *Nocardia* at the 80% amino acid sequence identity threshold. (**A**) The phylogenetic tree for the 87 species is shown on the left. The gene presence–absence matrix of 279,845 genes is shown on the right. In each row of the matrix, a gene presence is indicated by a blue dot and an absence by a white dot. (**B**) Pie chart for the numbers of gene clusters present in the core genome (873), shell genome (5,157), cloud genome (67,545), and new genes (205,970). (**C**) Unique vs new gene accumulation, showing the relationship between the unique genes (blue line) and new genes (orange line). (**D**) Gene accumulation curve, where the orange line represents the total number of genes in the pangenome and the blue line indicates the number of conserved homologous genes.

We performed a pangenome analysis using Roary for 87 *Nocardia* species (excluding *N. globerula*) at an 80% protein sequence identity threshold and found 279,845 gene clusters (see Materials and Methods). The core genome (99% ≤ species ≤ 100%; 502 clusters) represents the highly conserved set of genes across the genus and is likely essential for survival. The soft-core genome (95% ≤ species < 99%; 371 clusters) includes important genes and is nearly universal but may be absent in a few species (Fig. 3A and B). Together, core and soft-core genes total 873, representing broadly shared functional roles in *Nocardia* biology.

The accessory genome includes the shell genome (15% ≤ species < 95%; 5,157 clusters), reflecting more specialized genes, contributing to functional diversity, and enabling the host to adapt to different ecological conditions. These genes might provide genomic plasticity in ecological adaptation, allowing species within the genus to thrive in diverse environments. The cloud genome (2% ≤ species < 15%; 67,545 clusters) represents genes that occur infrequently and may confer specialized functions. The significant number of cloud genes shows the genetic variability within the genus and the potential for functional diversity across species. A key finding is 205,970 genes found exclusively in single species; they are considered new genes. This large number emphasizes frequent species-specific acquisitions (Fig. 3B and C). To distinguish between these categories, new genes are exclusive to one species, while unique genes are found in a few species but not widespread. As we add more genomes, there is an increase in unique genes, reflecting genomic diversity, while there is a decrease in new genes, indicating a decreasing chance of finding new genes or species-specific genes (Fig. 3C).

To quantify gene turnover across species, we calculated genomic fluidity (φ), as defined by (33). The average genomic fluidity of *Nocardia* was 0.76 ± 0.03, indicating that, on average, 76% of gene families differ between genome pairs, while only 24% remain shared. This high genomic fluidity confirms an open pangenome structure with extensive gene diversity (Fig. S3). The histogram of genomic fluidity values (Fig. S3) reveals a right-skewed distribution, reflecting the presence of a large accessory genome. Notably, the genomic fluidity in *Nocardia* is among the highest reported in bacteria. For comparison, *Pseudomonas fluorescens* exhibits a lower genomic fluidity of 0.41 (34), while *Streptomyces* species, known for their high biosynthetic potential, show a genomic fluidity of 0.51 (35). These values highlight the exceptional gene turnover and functional variability in *Nocardia*, reinforcing its ability to adapt to diverse ecological niches. To further assess genomic diversity, we computed pairwise Jaccard distances, which provide a gene content-based measure of dissimilarity between 87 species. The heatmap of Jaccard distance values demonstrated considerable heterogeneity among *Nocardia* species, indicating substantial variation in gene content across the genus (Fig. S4) (36, 37).

These observations suggest that *Nocardia* possesses an open pangenome, characterized by the continuous addition of new genes and the presence of highly divergent gene families as more species are included (Fig. 3D). In Fig. 3D, the total gene count is shown to increase as more species are added, while conserved gene count remains unchanged, confirming an open pangenome. The log-log plot of Heap’s Law further supports these findings, demonstrating a sublinear relationship (α = 0.80) between the cumulative unique genes and the number of genomes analyzed (Fig. S5). This α value below 1 confirms an open pangenome, where unique gene accumulation continues without saturation.

The Roary gene accumulation curve shows that while the discovery of unique genes continues, there is a reduction in new gene discovery , indicating saturation in species-specific gene acquisition. The high numbers of cloud and new genes point to ongoing genetic diversification, resembling patterns observed in other Actinomycetes, such as *Streptomyces* (35) and *Mycobacterium* (38)*,* where cyclic gene gain and loss enable species to specialize within overlapping or complex habitats. Overall, these patterns reflect the extensive genomic diversity within *Nocardia*, shedding light on how different species may have evolved to occupy various ecological niches and specialize in distinct functional roles.

In the above analysis, we excluded *N. globerula* because our phylogenetic analysis showed a much closer evolutionary relationship of *N. globerula* with *Rhodococcus erythropolis* than with other *Nocardia* species. Indeed, when it is included, the number of core genes is reduced from 502 to 273, a 54% reduction, which is unreasonably large. In our above analysis, the 80% sequence identity threshold was chosen to balance sensitivity and specificity, capturing conserved and moderately divergent genes across the highly diverged genus. For comparison, the pangenome results at the 85% threshold, both with and without *N. globerula*, are provided in Table S5.

### Functional profiling of annotated genes

**Fig 4:**
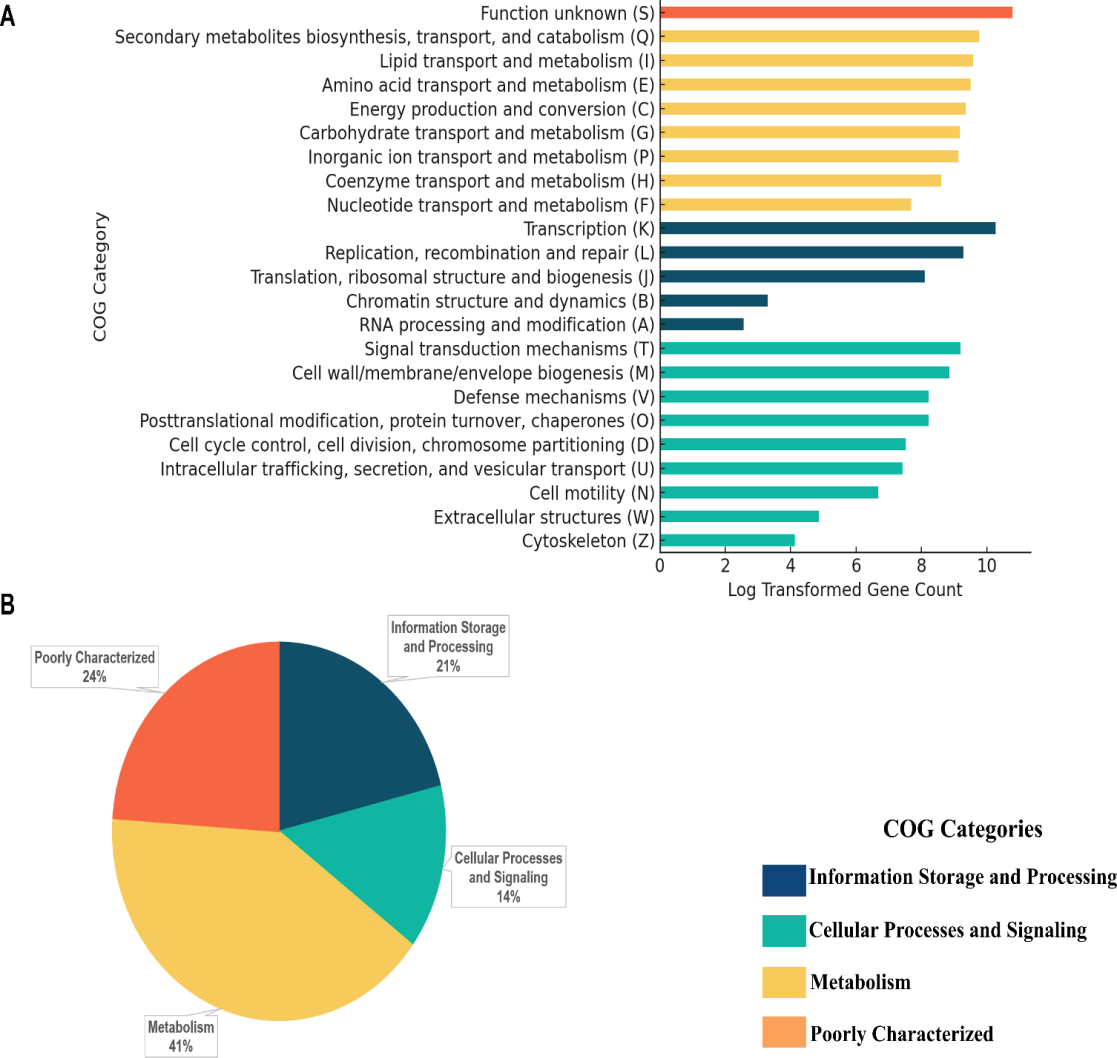
Functional annotation distribution in 87 *Nocardia* species. (**A**) The frequency distribution of COG categories in four functional groups: (i) information storage and processing; (ii) cellular processes and signaling; (iii) metabolism; and (iv) poorly characterized. The x-axis represents the occurrences of each functional category, and the values have been transformed using a natural logarithm (log1p) to better visualize both small and large gene counts. This transformation ensures that zero values are handled properly and that smaller values are spread out while larger ones are compressed, making it easier to observe differences between COG categories (such as A, B, Z, and W). (**B**) The pie chart for the distribution of functional groups includes information storage and processing, cellular processes and signaling, metabolism, and poorly characterized.

Our functional annotation of *Nocardia* genomes, using eggNOG-mapper v2 with the DIAMOND database, revealed that a significant proportion of *Nocardia* genes are related to transcription (12%) and secondary metabolism (7%), reflecting the genus’s high capacity for gene regulation and bioactive compound production. Compared to *Streptomyces*, where secondary metabolism genes account for only 2%–4% (39, 40), *Nocardia* genomes show a higher proportion of genes for bioactive compound synthesis (7%). Notably, a high proportion (38%) of genes are categorized as poorly characterized, with 20% classified as “function unknown” and 18% unassigned to any COG category. Interestingly, most uncharacterized genes (72%) are newly identified genes (Table S6; Fig. 4A and B), suggesting high potential for discovering novel functions. Our functional profiling efforts have reduced the proportion of hypothetical proteins from over 50% to 38%, similar to previous studies (18–21), which reported 20%–45% of genes unassigned to any function in *Nocardia*. Comparatively, *Streptomyces* genomes show a somewhat higher proportion (45%–70%) of proteins with unknown functions (37, 39). Our findings signify a step forward in functional annotation and provide a clearer genomic landscape of the genus.

The *Nocardia* core genome, essential for cellular maintenance and survival, is enriched in highly conserved genes involved in protein synthesis, metabolic regulation, and gene expression. This suggests that maintaining an efficient cellular machinery and genomic integrity is vital for *Nocardia* species to thrive in diverse environments and withstand environmental stressors (41, 42). In contrast, the accessory genome exhibits significant genetic variability, contributing to *Nocardia*’s adaptability through robust gene regulation, metabolic versatility, and environmental responsiveness. Key accessory genes are linked to secondary metabolite biosynthesis, lipid transport, amino acid metabolism, and energy production, which enables bioactive compound production, membrane stability, and nutrient utilization. The metabolic versatility of *Nocardia* species, including their ability to degrade hydrocarbons, complex organic compounds, and environmental pollutants, suggests a potential adaptation to nutrient-limited environments and diverse ecological niches (43–45) (Fig. S6). Their capacity to produce bioactive secondary metabolites and acquire essential nutrients such as iron further supports their ecological success and adaptability (46). However, further phenotypic studies are required to confirm these genomic predictions in specific environmental settings.

Interestingly, the core genome lacks genes for RNA processing, chromatin dynamics, cell motility, extracellular structures, and cytoskeleton. Instead, these genes are predominantly found in the accessory genome or newly acquired genes, suggesting niche-specific adaptations or recent evolutionary acquisitions. Our functional annotation categorized 1.5% (>2,500 genes) of the newly assigned genes into the COG V (defense mechanisms) category, encompassing genes associated with antibiotic resistance (e.g., beta-lactamases, aminoglycoside resistance proteins, and chloramphenicol phosphotransferases), restriction-modification systems (e.g., types I, II, and III restriction enzymes and N-6 DNA methylases), and multidrug efflux transporters (e.g., ABC-type transporters). These genes are predominantly classified as part of the accessory genome, suggesting their role in antimicrobial resistance and genomic defense. The presence of multiple antibiotic resistance determinants, including beta-lactamases and efflux pumps, is consistent with reports in previous studies on *Nocardia* species, which exhibit varying resistance patterns to multiple antibiotics, including sulfonamides, aminoglycosides, and β-lactams (47, 48). Notably, while these genes were newly identified within *Nocardia* genomes in our study, their functional roles in antibiotic resistance and genome protection have been well documented in Actinomycetota (49). However, further studies are needed to elucidate their specific contributions to *Nocardia*’s ecological adaptability and potential pathogenicity.

### *Nocardia*’s rich biosynthetic repertoire

**Fig 5:**
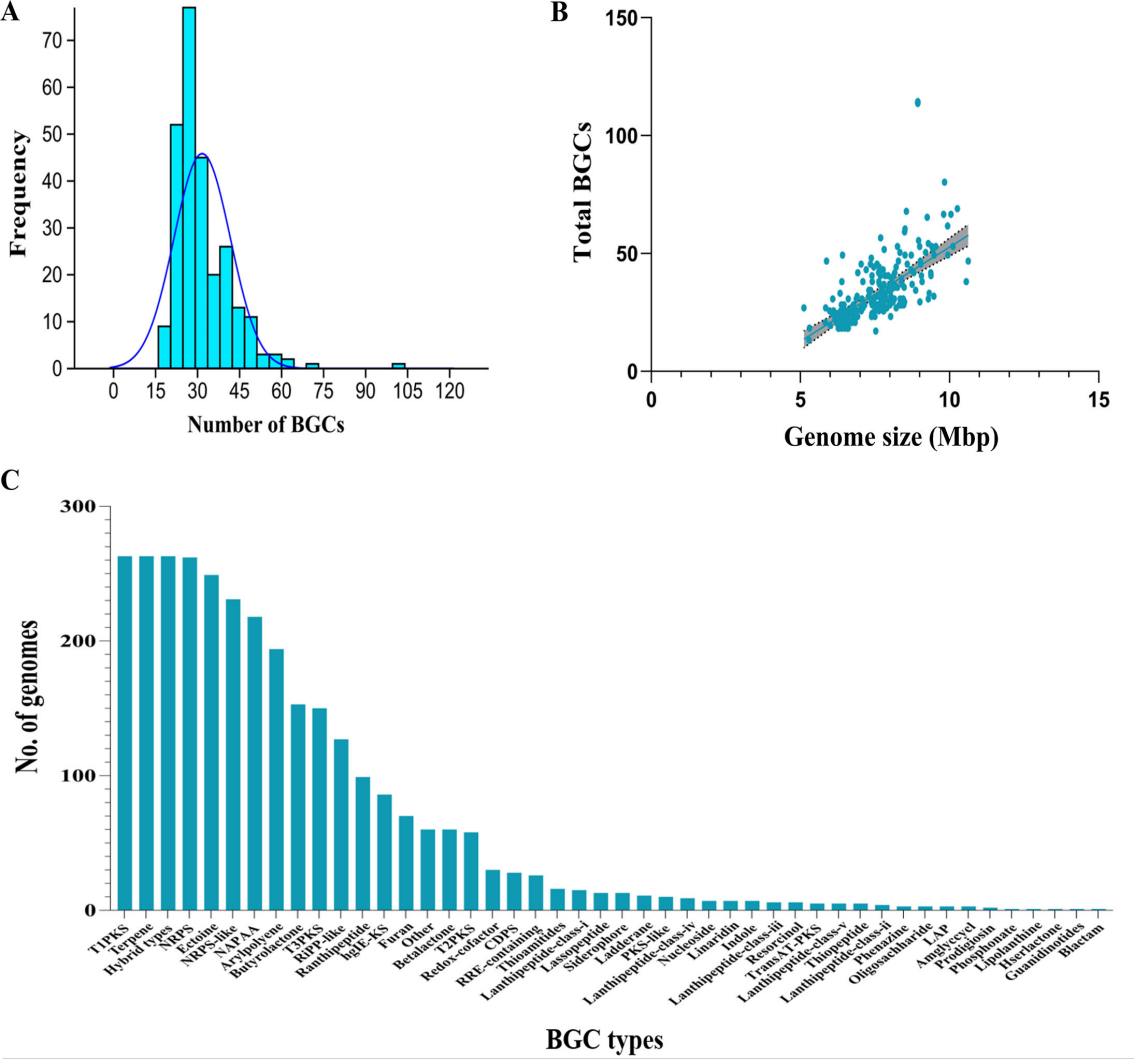
Biosynthetic gene cluster (BGC) profiling across *Nocardia* genomes. (**A**) Frequency distribution of total BGC counts per genome. (**B**) Scatter plot depicting the correlation between genome size and BGC count (PCC = 0.68, *P* < 0.0001). The shaded area represents the 95% confidence interval. (C) Distribution of the sizes of the 46 BGC classes in *Nocardia* genomes.

Our characterization of BGCs in *Nocardia* genomes revealed remarkable richness and diversity, showing a broad spectrum of biosynthetic capabilities within *Nocardia*. We identified 8,322 BGCs across 263 *Nocardia* genomes using antiSMASH v6.0 (see Materials and Methods), representing 46 different classes, ranging from 16 to 104 BGCs per genome (average: 34.92 ± 9.56). Approximately 35% (93 genomes) have 25–30 BGCs per genome, while ~5% (12 genomes) have >50 BGCs (Fig. 5A; Table S7A). A significant positive correlation (PCC = 0.68, *P* < 0.0001) is observed between BGC number and genome size, indicating that larger genomes tend to harbor more BGCs (Fig. 5B). For instance, *N. camponoti* CGMCC 4.7278 (5.21 Mbp genome) has 16 BGCs, while *N. tenerifensis* NBRC 101015 (9.73 Mbp genome) has 70 BGCs. The available JGI-IMG/MER data indicate that some species in Actinomycetota possess >100 BGCs, including *Amycolatopsis* kentuckyensis NRRL B-24129 (106 BGCs) and *Streptomyces sp*. ST1020 (114 BGCs). Our observation that *N. terpenica* NBRC 100888 possesses 104 putative BGCs has not been documented on *Nocardia* in MIBiG and JGI-IMG databases or in (10).

Large-scale genome mining studies (50, 51) have revealed the high biosynthetic potential of Actinomycetota, highlighting the prevalence of novel and uncharacterized BGCs. Therefore, we classified the identified BGCs into known (5,264 BGCs; 63.3%) and uncharacterized (3,058 BGCs; 36.7%) (Table S7A). This large proportion of known BGCs demonstrates that *Nocardia* harbors a diverse repertoire of biosynthetic pathways. However, the substantial presence of uncharacterized BGCs reveals a significant gap in our understanding of *Nocardia*’s biosynthetic capabilities. It further suggests the existence of unique biosynthetic pathways and novel chemical entities. Strain-specific variations further emphasize this untapped diversity. For instance, in *N. cyriacigeorgica*, strain BJ06-0127 has more uncharacterized BGCs (32) than known BGCs (13) (Table S7A). Such findings further suggest that *Nocardia* strains may specialize in producing strain-specific metabolites.

### Predominant BGC classes and evolutionary insights

Hybrid BGCs, i.e., those containing genes from distinct biosynthetic pathways within a single genomic region, are identified. They are cataloged into 248 types, with each genome averaging five hybrid BGCs. The presence of multiple biosynthetic pathways within these clusters suggests extensive functional diversity and potential for novel bioactive compound production, consistent with previous findings (52, 53). Predominant hybrid BGCs include NRPS-terpene (179 genomes), ribosomally synthesized and post-translationally modified peptides-like (NRPS-RiPP; 164 genomes), NRPS-NRPS-like (83 genomes), and T1PKS-NRPS (70 genomes), suggesting diverse combinations of biosynthetic pathways (Fig. 5C; Table S7B). Predominant BGC classes include type 1 polyketide synthase (T1PKS), terpenes, and non-ribosomal polypeptide synthetases (NRPS) universally present across all genomes. Other common classes include ectoine (249 genomes), NRPS-like fragment (231 genomes), and non-alpha poly-amino acids (NAPAA) like e-polylysin (218 genomes) (Table S7B). These six BGC classes collectively contribute ~65% to the total BGCs currently found in *Nocardia* genomes. Five types of PKS (T1PKS, T2PKS, T3PKS, PKS-like, and trans-AT PKS) contribute 17.5% of all the BGCs. Also, NRPS and NRPS-like (NRPS-like fragments) together constitute about 30.4% of the total BGCs. RiPP-related BGCs (5% of total) include ranthipeptide, RiPP-like, thioamitides, lanthipeptide class (i to v), lassopeptide, linear azol(in)e-containing peptides (LAP), RiPP recognition element (RRE)-containing, linaridin, lipolanthine, thiopeptide, and guanidinotides. Interestingly, uncharacterized lanthipeptide-class-v BGC is found in five genomes of three species (*N. elegans*, *N. mexicana,* and *N. vulneris*), marking its first report in *Nocardia*. Further examination reveals no sequence similarity between the lanthipeptide-class-v BGCs and any entries in the MIBiG database, suggesting a potentially novel bioactive compound. Also, the prodigiosin BGC is found in two genomes of *N. salmonicida*, with 40%–45% gene sequence similarity with BGC0001063 from the MIBiG database, which encodes undecylprodigiosin (54, 55). This finding is significant as it marks the first report of the prodigiosin BGC in *Nocardia*, revealing the genus’s potential for producing prodigiosin-like compounds that possess antimicrobial and anticancer properties.

**Fig 6:**
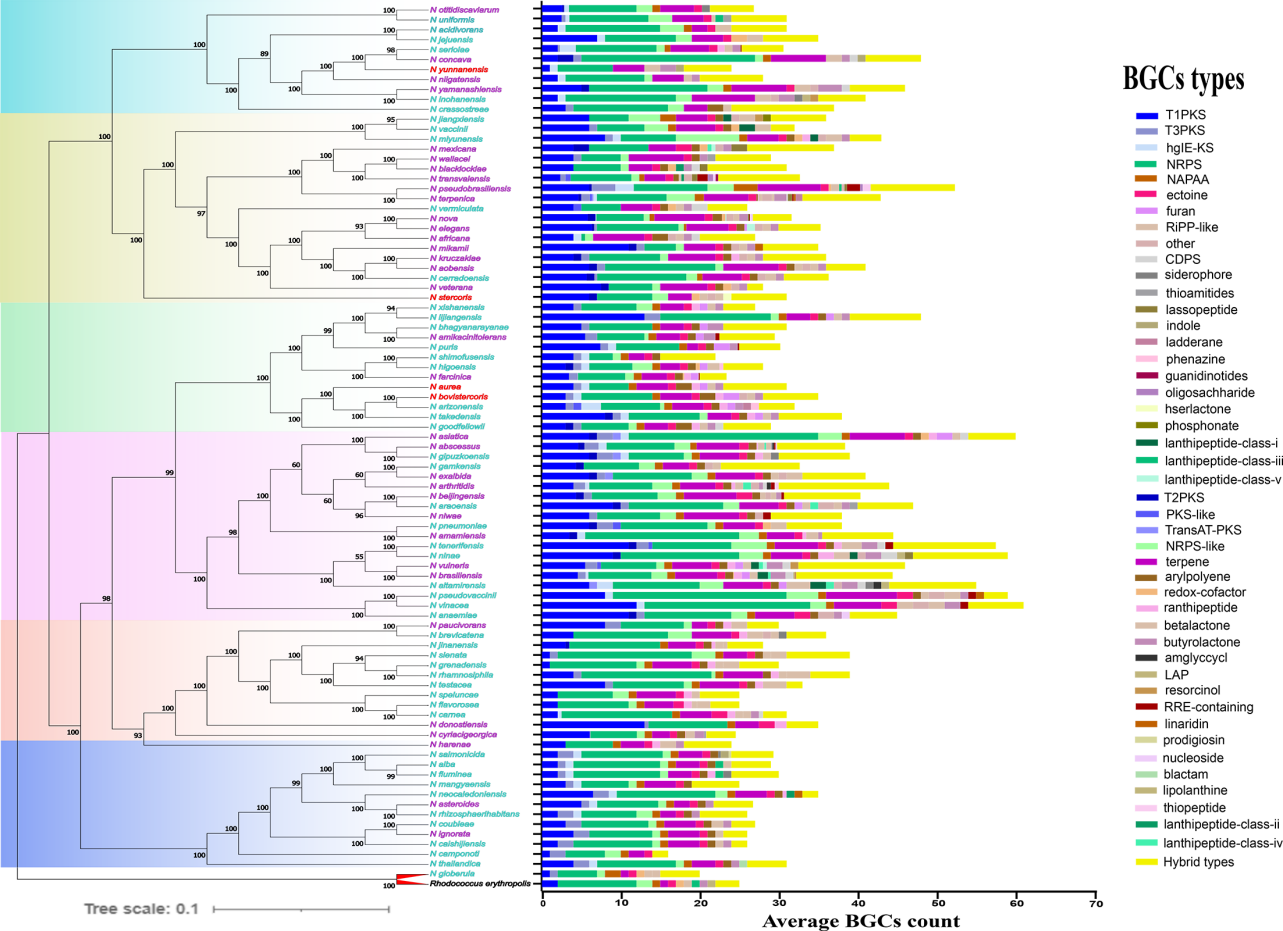
Evolutionary dynamics of the 46 inferred BGC classes in the genus *Nocardia*. The phylogenetic tree is color-coded to highlight the six major phylogroups (clades), and the *Nocardia* species are color-coded based on pathogenicity, as mentioned in Fig. 2. The bar graph to the right of the phylogenetic tree shows the average number of BGCs per species.

The distribution of 46 BGC classes across 88 *Nocardia* species was examined to understand their evolutionary relationships. T1PKS, NRPS, and terpenes are conserved across all *Nocardia* species, suggesting their widespread retention due to functional significance rather than lineage-specific inheritance (Fig. 6). Similar patterns have been previously observed in *Nocardia* (56) and other Actinomycetota such as *Streptomyces* (57) and *Kutzneria* (58), as well as in broader Actinomycetes studies (59), where these three BGCs are widespread and play key roles in secondary metabolism and adaptation. BGC counts vary significantly, with an average of 35 BGCs/species, with Clade VI species having the highest (47) and Clade V the lowest (27). The extensive genomic plasticity and the diverse biosynthetic potential of *Nocardia* highlight their remarkable adaptability to various environmental conditions. Variation in BGC counts across clades suggests that gene gain and loss events have often occurred in the evolution of *Nocardia*. This observation aligns with findings in other bacteria (60–62). Clade-specific variations further emphasize the evolutionary pressures, such as the presence of all lanthipeptide classes (I to V) and oligosaccharide BGCs in Clade II, while the confinement of amglyccycl BGCs to Clade IV species was observed. The abundance of species-specific and accessory genes highlights the role of gene turnover in shaping microbial genomes and facilitating niche differentiation. Additionally, the production of diverse secondary metabolites may influence microbial competition, symbiosis, and nutrient cycling, ultimately shaping ecosystem functions (63, 64).

### Network complexity and potential interconnections of *Nocardia* BGCs

**Fig 7:**
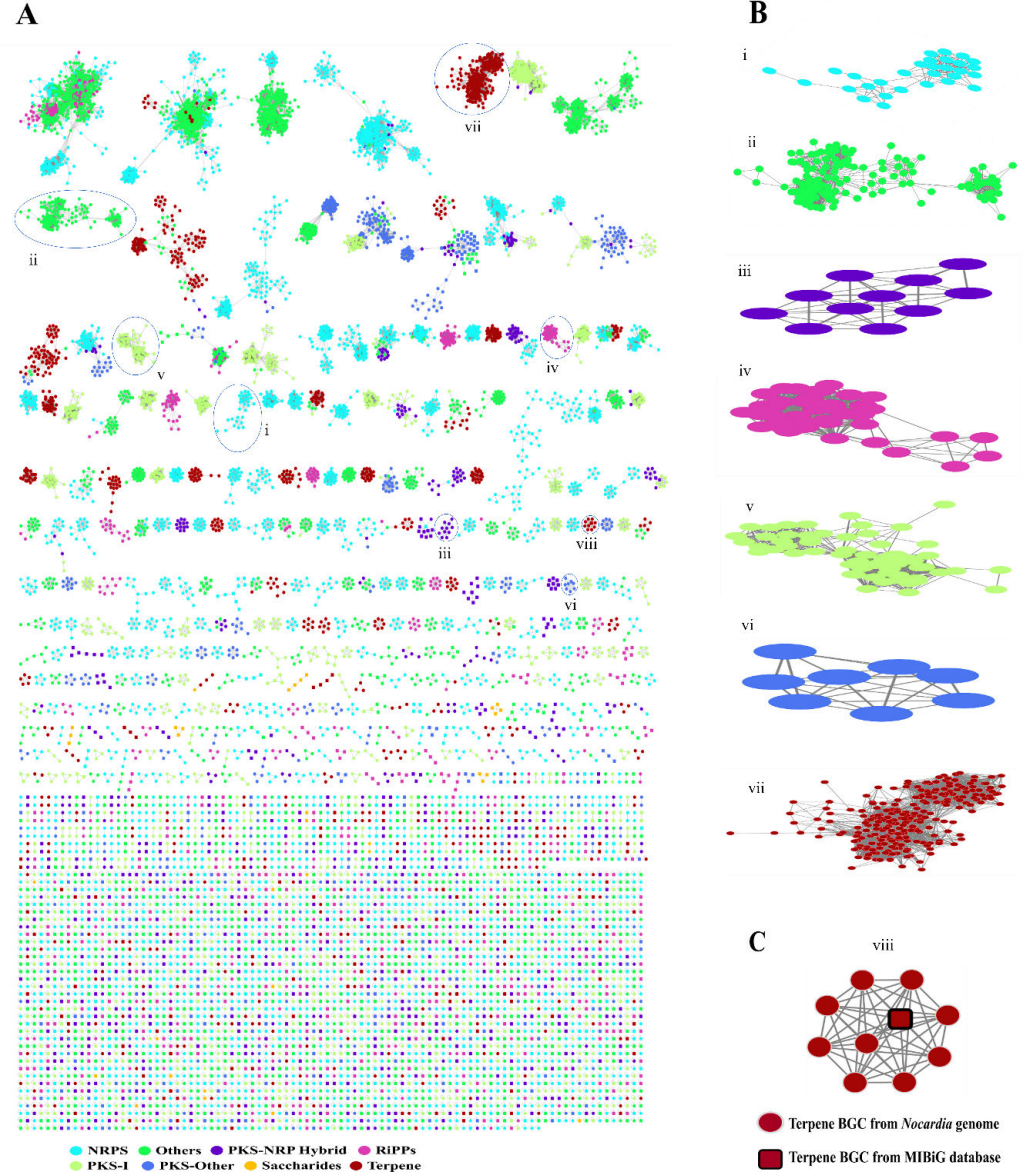
Sequence similarity networks (SSNs) of BGCs. (**A**) BiG-SCAPE cluster-type sequence similarity networks of 10,819 BGCs, including 8,322 inferred BGCs from the analyzed *Nocardia* genomes and 2,497 BGCs from the MIBiG database. Colors show different types of GCF categories; see the color symbols under the figure. (**B**) Zoom-in of the GCF network for each BiG-SCAPE class, excluding saccharides, hybrid BGCs, and the BGCs from MIBiG. This zoom-in highlights the dense networks of GCFs within each BiG-SCAPE class. Most of the analyzed saccharides are singletons, and the remaining saccharides GCFs shown in Fig. 7A are BGCs from MIBiG, which are not included in this zoom-in section. (**C**) Zoom-in of the terpene GCF network in panel A, along with the information of the BGCs from MIBiG.

Using BiG-SCAPE, we constructed a similarity network of 8,322 *Nocardia* BGCs and 2,497 reference BGCs from the MIBiG database with a 0.3 protein sequence similarity cutoff to infer a broader network of relationships among the BGCs (see Materials and Methods). The constructed network comprises 10,819 nodes (BGCs) and 76,693 edges (connections), indicating a diverse array of BGCs with shared gene content or structural similarities (Fig. 7A; Table S8A). On average, each BGC connects to nearly 14 others (average node degree: 13.6), suggesting functional or evolutionary connections. The gene cluster families (GCF) network for each BiG-SCAPE class, excluding saccharides, hybrid BGCs, and BGCs from MIBiG, reveals dense networks within each class (Fig. 7B). The majority of analyzed saccharides are singletons, and the saccharides shown in panel A are from MIBiG. Detailed analysis of terpenes GCFs, including MIBiG BGCs, reveals intricate relationships and potential novel pathways (Fig. 7C). The network includes 4,080 GCFs, many of which are isolated, and 3,220 singletons, reflecting unique or less common BGCs with potential novel biosynthetic capabilities. Such a large number of GCFs and singletons highlights the network’s complexity and the uniqueness of many BGCs, as seen in other Actinomycetes (50, 51). Class-specific connectivity reveals NRPS as the most abundant (1,933 GCFs, 4,929 BGCs, and 1,513 singletons) (Fig. S7; Table S8B), while saccharides are least represented (73 GCFs, 113 BGCs, and 64 singletons).

Among the 2,497 MIBiG BGCs, only 11 are attributed to *Nocardia* genomes, with classes like NRPS and PKS-other contributing the highest counts (Table S8B). Interestingly, no *Nocardia* BGCs have been deposited in the PKS-I and saccharides categories, highlighting gaps in the representation of *Nocardia* BGCs in the MIBiG database. GCFs identified in this study, such as NRPS and T1PKS NRPS, possess the most MIBiG reference BGCs, suggesting that *Nocardia-*derived BGCs in these clusters have the potential to produce compounds similar to those associated with MIBiG BGCs (Fig. S8). This also indicates their evolutionary and functional relevance in producing compounds with known bioactivities. In contrast, GCFs such as siderophores and lanthipeptide class-I had fewer reference BGCs, indicating more specialized or less common biosynthetic potential. These findings demonstrate that *Nocardia* harbors both conserved and uncharacterized biosynthetic pathways, with isolated clusters and singletons offering significant potential for novel product discovery.

### Synteny and BlastP analysis of genes for antitumor and anticancer compounds in Actinomycetota

We conducted thorough synteny and BlastP analyses of gene clusters associated with three bioactive compounds (nocobactin NA, nargenicin, and brasilicardin A) (see Materials and Methods for details). These integrated analyses identified novel *Nocardi*a strains that may serve as new sources of bioactive compounds and provided insights into their evolutionary history.

### Nocobactin NA gene cluster

**Fig 8:**
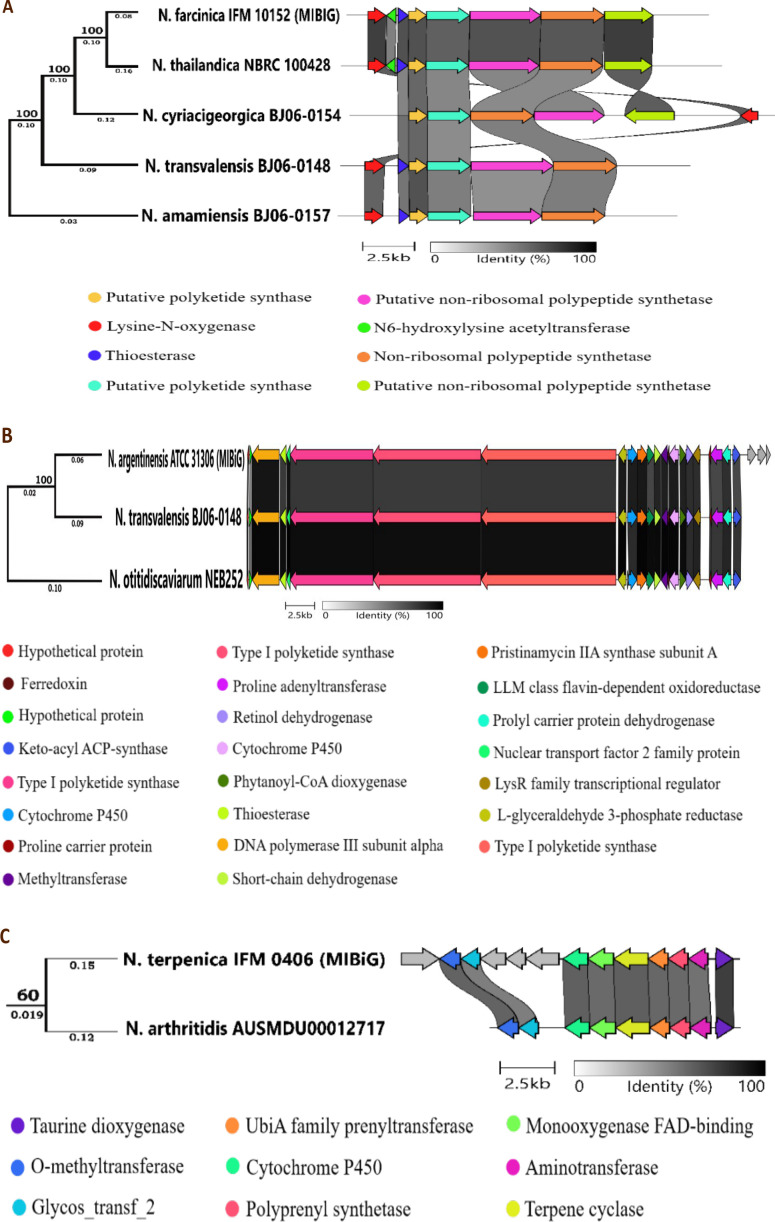
Synteny analysis of the (**A**) nocobactin NA gene cluster; (**B**) nargenicin gene cluster; and (**C**) brasilicardin A gene cluster. Comparison of inferred syntenies of the genes in a BGC in *Nocardia* genomes, using the reference synteny in MIBiG. The results of our synteny analysis of three gene clusters (BGCs) are shown in three separate figures. In each figure, homologous coding sequences (CDSs) are color-coded and connected by grayscale bars, indicating amino acid identity percentages (right panel). Only genes showing similarity to the MIBiG reference cluster (the top cluster in each figure) are included. The phylogenetic relationships of the *Nocardia* genomes with a BGC similar to the reference gene cluster from MIBiG are shown in the left panel. A bootstrap value is shown at each branching node, and branch lengths, representing the number of substitutions per site, are shown below the branches.

Nocobactin NA, an iron-chelating NRPS-type siderophore, enhances *Nocardia*’s ability to scavenge iron, contributing to its pathogenicity and cytotoxic, antitumor, and antimuscarinic activities (12, 17, 65). Identified as *BGC0001027* in *N. farcinica* IFM 10152 in the MIBiG database, the cluster includes eight genes, including three core (essential) NRPS genes. It is detected in 45 genomes of 5 *Nocardia* species: *N. farcinica* (41 genomes) and one genome for each of *N. thailandica*, *N. cyriacigeorgica*, *N. transvalensis*, and *N. amamiensis*. Genomes of *N. farcinica* were excluded from our analysis because they exhibited 100% similarity with the reference gene cluster. *N. thailandica* NBRC 100428 has retained all eight genes, indicating full capacity. *N. cyriacigeorgica* BJ06-0154 exhibits the presence of all core genes, while one of the NRPS genes is in reverse orientation and the lysine-N-oxygenase is invertedly translocated from the 5′ end to the 3′ end of the cluster. Both *N. transvalensis* BJ06-0148 and *N. amamiensis* BJ06-0157 lack core genes, potentially disabling Nocobactin NA production (Fig. 8A). The cluster is confined to Actinomycetota, distributed across seven genera, suggesting an evolutionary origin from a common ancestor. The exclusive presence of lysine-N-oxygenase in *Nocardia* highlights its central role in the evolution and production of Nocobactin NA. While its distribution across species suggests possible HGT or independent evolutionary retention, further analyses are needed to confirm the underlying mechanisms (Table S9A).

### Nargenicin gene cluster

Nargenicin, a macrolide polyketide with an oxa-bridged decalin core, exhibits antibiotic, anticancer, and cytotoxic activities (12, 66). Identified as *BGC0002024* in the MIBiG database, this cluster in *N. argentinensis* ATCC 31306 includes 26 genes, which include four core genes (three belonging to T1PKS and one to keto-acyl ACP-synthase). Sequence similarity to the reference cluster is found in *N. otitidiscaviarum* NEB252 and *N. transvalensis* BJ06-0148. Both species have retained 23 of the 26 genes, including all core genes, suggesting their capacity to produce similar compounds. The pathway appears conserved across these species despite minor genetic divergence, with no gene rearrangements observed (Fig. 8B). These genes are primarily distributed within Actinomycetota, with core genes shared with *Streptomyces*, suggesting a common evolutionary origin. The GGDEF domain-containing protein unique to *Nocardia* suggests genus-specific adaptation (Table S9B).

### Brasilicardin A gene cluster

Brasilicardin A, an isoprenoid-hybrid compound with immunosuppressive, cytotoxic, and antitumor activities (12, 17, 67), is biosynthesized by the gene cluster BGC0000632 in *N. terpenica* IFM 0406. This cluster includes 13 genes, including one core gene (terpene). This cluster shows sequence similarity with nine genomes, with eight genomes in *N. terpenica* and one genome in *N. arthritidis* AUSMDU0001271. Genomes of *N. terpenica* were excluded from our analysis because they are 100% similar to the reference cluster. *N. arthritidis* AUSMDU0001271 has retained 9 of the 13 genes, including the core gene (terpene cyclase) but lacks the regulatory gene (transcriptional regulator), suggesting differences in biosynthetic regulation (Fig. 8C). No gene translocations or changes in gene orientations are observed. Genes are predominantly found within Actinomycetota, with the terpene cyclase gene also found in *Blastocatellia* (Acidobacteriota) and AMP-dependent synthetase genes in *Blastococcus* (Bacillota), indicating potential HGT across different phyla. The higher prevalence of these genes in *Streptomyces* compared to *Nocardia* suggests potential evolutionary changes, including gene transfer or differential gene retention, within Actinomycetota (Table S9C).

### Conclusions and perspectives

Overall, our study provides insights into multiple facets of the *Nocardia* genus, including its pangenome structure, pathogenicity, evolutionary relationships, and biosynthetic potential. By uncovering its open pangenome, high genomic plasticity, high genomic fluidity, and diverse BGCs, we highlight the genus’s remarkable adaptability and potential for secondary metabolite discovery. Our phylogenomic and pangenome analyses revealed that *Nocardia* species exhibit substantial gene turnover, driven by gene gain, gene loss, and HGT, that shape microbial evolution and ecological adaptation. While HGT is often implicated in bacterial genome evolution, our study did not include a formal HGT analysis. Thus, the role of gene loss, gene retention, and independent evolutionary events should also be considered in explaining the species-species differences in *Nocardia*. By elucidating the biosynthetic capabilities of *Nocardia*, this study has enhanced our knowledge of microbial interactions, niche specialization, and the potential impact of these bacteria on their environments. While our study has broadened the understanding of *Nocardia*’s genomic landscape, several knowledge gaps remain. Notably, the absence of experimental validation limits our findings, and future studies should aim to verify the bioactivity of the identified uncharacterized BGCs and their associated secondary metabolites. Experimental approaches, such as gene expression analysis and metabolomic profiling, will be required to determine the functional expression and metabolite production of these BGCs.

Further exploration of *Nocardi*a’s genomic and functional diversity, alongside enhanced sampling strategies and genomic data completeness, will be needed to unlock its full biosynthetic potential and ecological roles. Expanding genomic sampling and experimental characterization will not only validate our computational findings but also provide novel insights into *Nocardia*’s capabilities in natural product discovery. The limited representation of *Nocardia* BGCs in databases like MIBiG highlights opportunities for further cataloging. This study establishes *Nocardia* as a valuable bacterial genus for novel secondary metabolites with potential biotechnological and pharmaceutical applications. Continued research into its diverse functions across different environments, combined with experimental efforts, will increase *Nocardia’*s position as a valuable source of novel secondary metabolites.

## MATERIALS AND METHODS

### Genome collection, quality assessment, and annotation

From the NCBI GenBank, 303 genome assemblies of *Nocardia* were obtained as of 29 July 2021. Given the taxonomic complexities in the *Nocardia* genus, only genomes corresponding to species with “validly published*”* status in the LPSN database (68) (https://www.bacterio.net/) were included. This resulted in 263 genomes, representing 88 taxonomically recognized species, which were subjected to downstream analysis. Genome assemblies were assessed for quality and completeness using BUSCO v5.1.2 with the *Corynebacteriales_odb10* lineage data set in the genome mode (69). Prokka v1.4.6 was used to reannotate the genomes (70).

### Classification of pathogenicity and phylogenetic analysis of *Nocardia* species

*Nocardia* species were classified into pathogenic and non-pathogenic, using published literature, and online databases (LPSN [https://lpsn.dsmz.de/genus/Nocardia]**,** GLoBI [https://www.globalbioticinteractions.org/], and BacDive [https://bacdive.dsmz.de/]). Species were categorized as pathogenic if they had been reported in multiple independent human or animal infections in peer-reviewed studies. Also, based on their severity and impact on human health, risk group levels were assigned as per the LPSN database, categorizing pathogenic species into two risk groups: group 1 (unlikely to pose significant threats to human health) and risk group 2 (posing a high risk to human health).

We performed genome-wide average nucleotide identity (ANI) analysis using FastANI v1.34 (23) to compute pairwise genomic similarity among the given genomes, comparing all genomes against each other and generating a formatted ANI distance matrix. We used ANIclustermap v1.4.0 with the fastani mode for clustering and visualization (24) (https://github.com/moshi4/ANIclustermap). To further understand the evolutionary relationships among these classified species, a consensus genome was created for each species and aligned using MUSCLE v5.1 (71), making sure that each position has at least 20% sequences that have no gaps. This resulted in 88 high-quality genomes. OrthoFinder v2.5.4 (72) with default parameters was utilized to find the single-copy orthologous genes, which were concatenated for phylogenetic analysis. From the identified orthogroups, 110 single-copy orthologous genes were chosen for phylogenetic reconstruction to ensure phylogenetic accuracy, avoid paralogs, and ensure an accurate evolutionary signal. Mafft v7.505 (73) was used to align the protein sequences, and *Rhodococcus erythropolis* NBRC 15567 was used as the outgroup. This species was chosen as an outgroup due to its taxonomic placement within the Corynebacteriales order, closely related to *Nocardia* yet evolutionarily distinct, making it an appropriate reference for rooting the phylogenetic tree. A maximum likelihood tree using the GTR + I + G model as the selected model of protein sequence evolution was constructed via IQTREE v2.2.0.3 (74). Bootstrap values were calculated from 1,000 replicates using an ultrafast bootstrap method. MEGA X (75) was used to visualize and edit the tree. In addition, patristic distances between species were computed using Geneious Prime v.2022.2.2 with default parameters to examine evolutionary divergence.

### Pangenome analysis and functional annotation

Pangenome analysis was performed using Roary (76), with Prokka-derived GFF3 files as initial input. To ensure consistent gene cluster representation for species with multiple genomes, we combined multiple GFF files from all genomes of each species, selecting the best gene representative for each cluster based on the longest chromosome. Genes outside predefined clusters were identified as unclustered. This generated unified GFF3 files for each species, which were then used as input for Roary. Gene content was compared across species at an 80% protein sequence identity threshold, capturing both conserved and moderately divergent genes. An alternative threshold of 85% was used for comparison. To quantify genome-level dissimilarity and evaluate the openness of the *Nocardia* pangenome, we calculated the genomic fluidity (φ) following the method described by (33). This metric estimates the proportion of unique gene families relative to the total gene families in a given genome pair. To quantify gene content dissimilarity among *Nocardia* species, we computed pairwise Jaccard distances using the gene presence–absence matrix derived from Roary. The Jaccard distance measures the proportion of shared gene families between two species relative to their total unique gene content, providing a quantitative assessment of genome similarity. The resulting Jaccard distance matrix was visualized as a heatmap. We applied Heap’s law to quantitatively assess the openness or closeness of the pangenome. Heap’s law is formulated as P = k·N^α^, where P represents the cumulative number of genes, N is the number of genomes analyzed, and k and α are parameters to be determined through non-linear regression. In our approach, the parameter α indicates the degree of pangenome openness; that is, if α < 1, the pangenome is open, and if α > 1, the pangenome is closed (31, 32).

For functional annotation, eggNOG 5.0 (77) and eggNOG-mapper v2 (78) (http://eggnog5.embl.de/#/app/home) were used to predict the clusters of orthologous genes (COGs) in 87 species with the DIAMOND mapping mode and taxonomy scope in bacteria.

### BGC detection and network analysis

BGCs were detected in the 263 *Nocardia* genomes using antiSMASH v6.0 (79), with relaxed detection strictness. Hybrid BGCs were identified based on the presence of genes from two or more distinct biosynthetic pathways within a single genomic region. This classification was based on antiSMASH annotations, where overlapping or adjacent BGC features indicated multiple biosynthetic types. To assess the relationship between the total BGCs and genome size in each genome, we performed a Pearson correlation analysis. The Pearson correlation coefficient (PCC) and the two-tailed *P*-value were calculated, with statistical significance evaluated based on the obtained *P*-value and the 95% CI. Gene clusters derived were compared with the MIBiG (v3.0) database (80) and classified into two groups: known BGCs and uncharacterized BGCs. Known BGCs were defined as BGCs that exhibit similarity with gene clusters or compounds documented in the MIBiG database, whereas uncharacterized BGCs lack similarity with any MIBiG entries. Also, 2,497 BGCs from the MIBiG database were included in the resulting putative BGCs derived from this study, and the final BGC set was used to construct BGC family networks, using BiG-SCAPE v1.1.5 (81). The command includes singleton BGCs (*--*include_singletons), considers hybrid BGCs (*--*mix), and operates in the auto mode (*--*mode auto), and several cutoffs (0.3, 0.5, 0.7, and 0.9) were tested for clustering (*--*cutoffs). Sequence similarity matrices derived from BiG-SCAPE were visualized using Cytoscape v3.10.1 (82).

### Synteny analysis of bioactive compound gene clusters and Blastp screening beyond *Nocardia*

We analyzed the synteny of the gene clusters associated with three known bioactive compounds (nocobactin NA, nargenicin, and brasilicardin A) derived from *Nocardia* genomes deposited in the MIBiG database. Secondary metabolite BGCs were identified using antiSMASH, while BiG-SCAPE (similarity cutoff: 0.5) and Clinker (sequence identity threshold: 50%) (83) were used for synteny mapping and gene cluster network analysis, respectively. This aided in identifying *Nocardia* BGCs similar to MIBiG reference BGCs and comparing their gene cluster arrangements. BlastP was performed on genes within these reference clusters using the NCBI non-redundant (nr) database to determine whether these biosynthetic genes are unique to the *Nocardia* genus or are also found in other genera or phyla.

## Data Availability

The data supporting the findings of this study, including gene annotation files, pangenome analysis outputs generated from Roary, secondary metabolite BGCs predictions from antiSMASH, and network analysis produced via BiG-SCAPE, as well as the custom Python scripts utilized in the data processing and analysis, will be made available upon request to the corresponding author.

## SUPPLEMENTAL MATERIAL

Supplemental figures mbio.00947-25-s0001.docx Figures S1 to S8.

Supplemental tables mbio.00947-25-s0002.xlsx Tables S1 to S9.

ASM does not own the copyrights to Supplemental Material that may be linked to, or accessed through, an article. The authors have granted ASM a non-exclusive, world-wide license to publish the Supplemental Material files. Please contact the corresponding author directly for reuse.
