# Supplementary material for: Nocardia genomes are a large reservoir of diverse gene content, biosynthetic gene clusters, and species-specific genes
Source: mBio. 2025 May 23;16(6):e00947-25. doi: 10.1128/mbio.00947-25 (PMC12153302; doi:10.1128/mbio.00947-25)
Supplement: Supplemental figures — Figures S1 to S8. [file mbio.00947-25-s0001.docx]

**Supplementary Figures (1-8)**

*Nocardia* Genomes are a Large Reservoir of Diverse Gene Content, Biosynthetic Gene Clusters, and Species-specific Genes

Kiran Kumar Eripogu^a,b,c^, Chun-Ping Yu^b^, An-I Tsai^b^, Jinn-Jy Lin^d^, Hsiao-Ching Lin^e^, Wen-Hsiung Li^a,b,f#^

**Affiliations:**

a. Biodiversity Program, Taiwan International Graduate Program, Academia Sinica, Taipei, 11529, Taiwan.

b. Biodiversity Research Center, Academia Sinica (BRCAS), Taipei, 11529, Taiwan.

c. Department of Life Sciences, National Taiwan Normal University (NTNU), Taipei, 11677, Taiwan.

d. National Center for High-performance Computing, National Applied Research Laboratories, Hsinchu, 30092, Taiwan.

e. Institute of Biological Chemistry, Academia Sinica, Taipei, 11529, Taiwan.

f. Department of Ecology and Evolution, University of Chicago. Chicago, IL 60637, USA.

**Corresponding Author:**

Wen-Hsiung Li, Department of Ecology and Evolution, University of Chicago. Chicago, IL 60637, USA. E-mail: [whli@uchigao.edu](mailto:whli@uchigao.edu)

**FIGURE S1** Genome annotation of *Nocardia* genomes.

**FIGURE S2** Average Nucleotide Identity (ANI) heatmap.

**FIGURE S3** Genomic fluidity (φ) distribution in *Nocardia*.

**FIGURE S4** Pairwise Jaccard distance heatmap of *Nocardia* species.

**FIGURE S5** Log-log plot for Heap's Law in *Nocardia* pangenome analysis.

**FIGURE S6** COG category distribution across core genome, shell genome, cloud genome, and new genes.

**FIGURE S7** Distributions of gene cluster families (GCFs) and singletons.

**FIGURE S8** MIBiG references BGC count across GCFs.

**
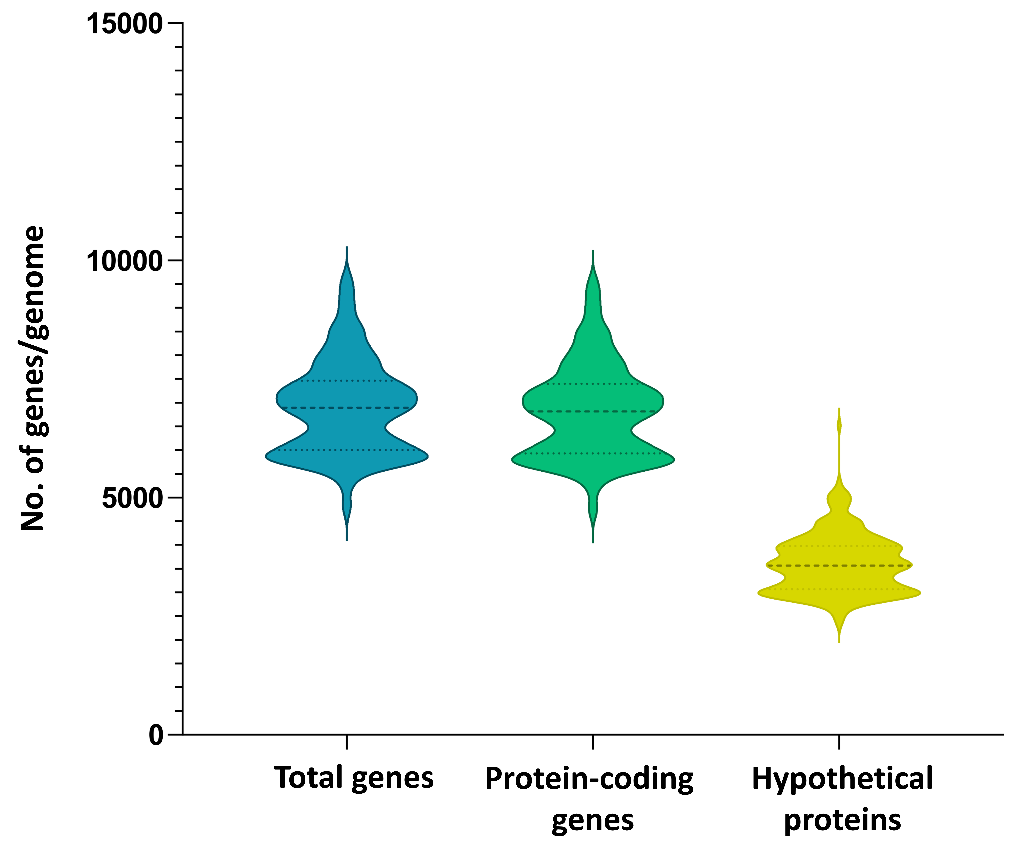
**

**FIGURE S1.** Genome annotation of *Nocardia* genomes using Prokka. Violin plots showing the distribution of total genes, protein-coding genes, and hypothetical proteins across all 263 *Nocardia* genomes. The X-axis represents gene categories (total genes, protein-coding genes, and hypothetical proteins), and the Y-axis represents the number of genes identified per genome.


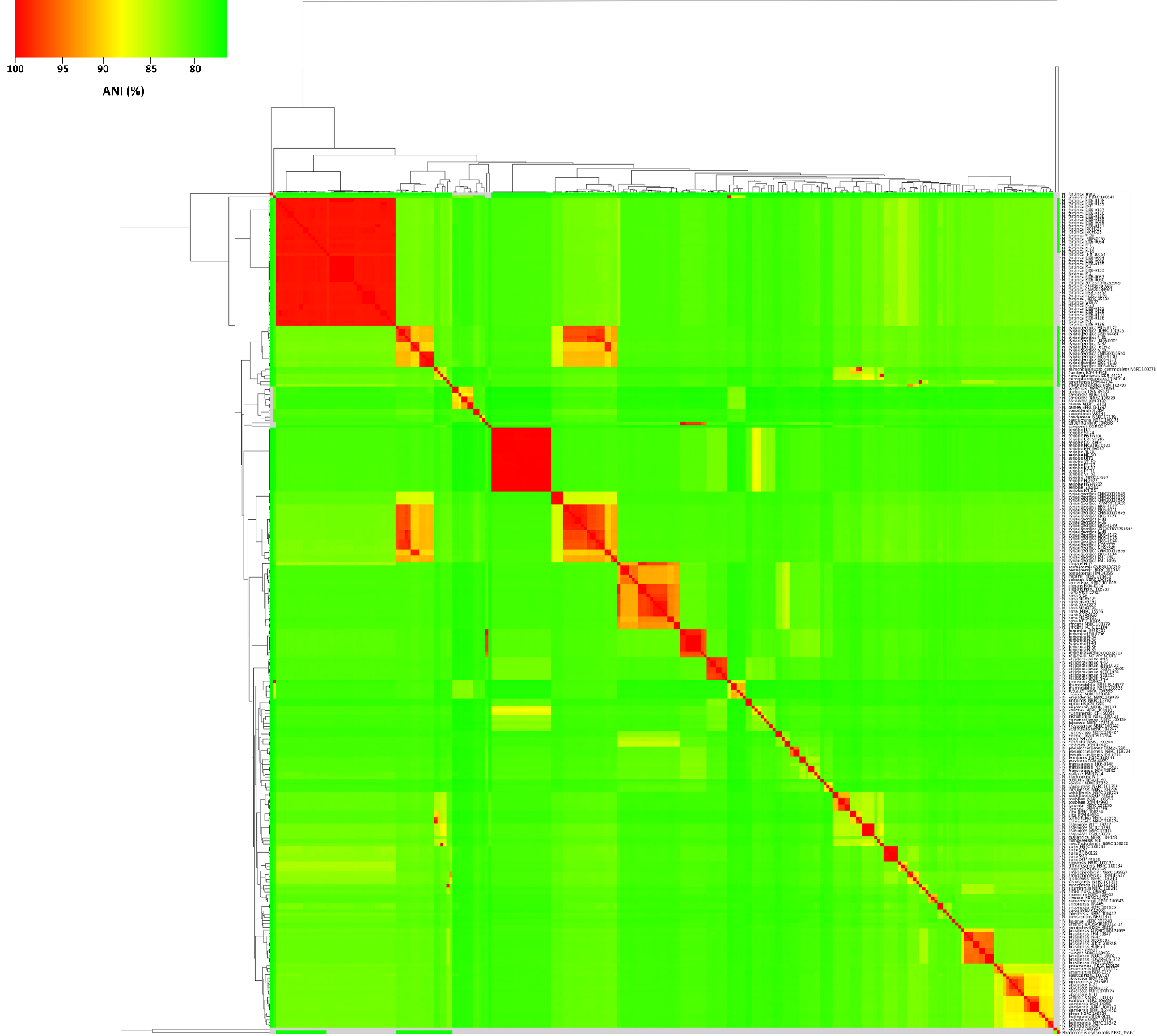


**FIGURE S2.** Average Nucleotide Identity (ANI) heatmap. This heatmap visualizes the ANI values among 263 *Nocardia* genomes and the outgroup *Rhodococcus erythropolis* NBRC 15567. The color gradient from green (low ANI) to red (high ANI) with hierarchical clustering.


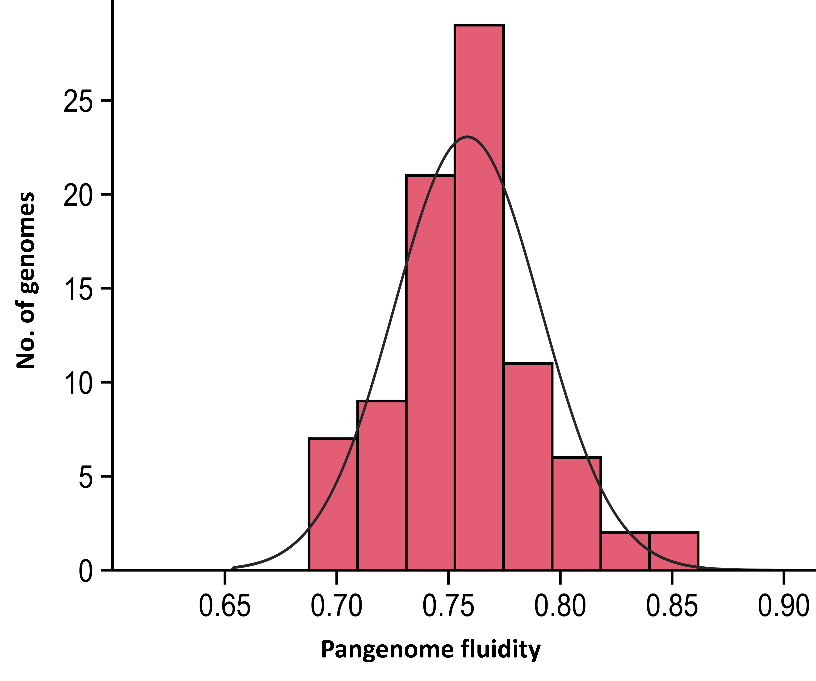


**FIGURE S3.** Genomic fluidity (φ) distribution in *Nocardia*. This histogram represents the genomic fluidity distribution in *Nocardia* species, where the x-axis denotes genomic fluidity values, indicating genome-level dissimilarity, while the y-axis represents the number of species exhibiting a given fluidity value.


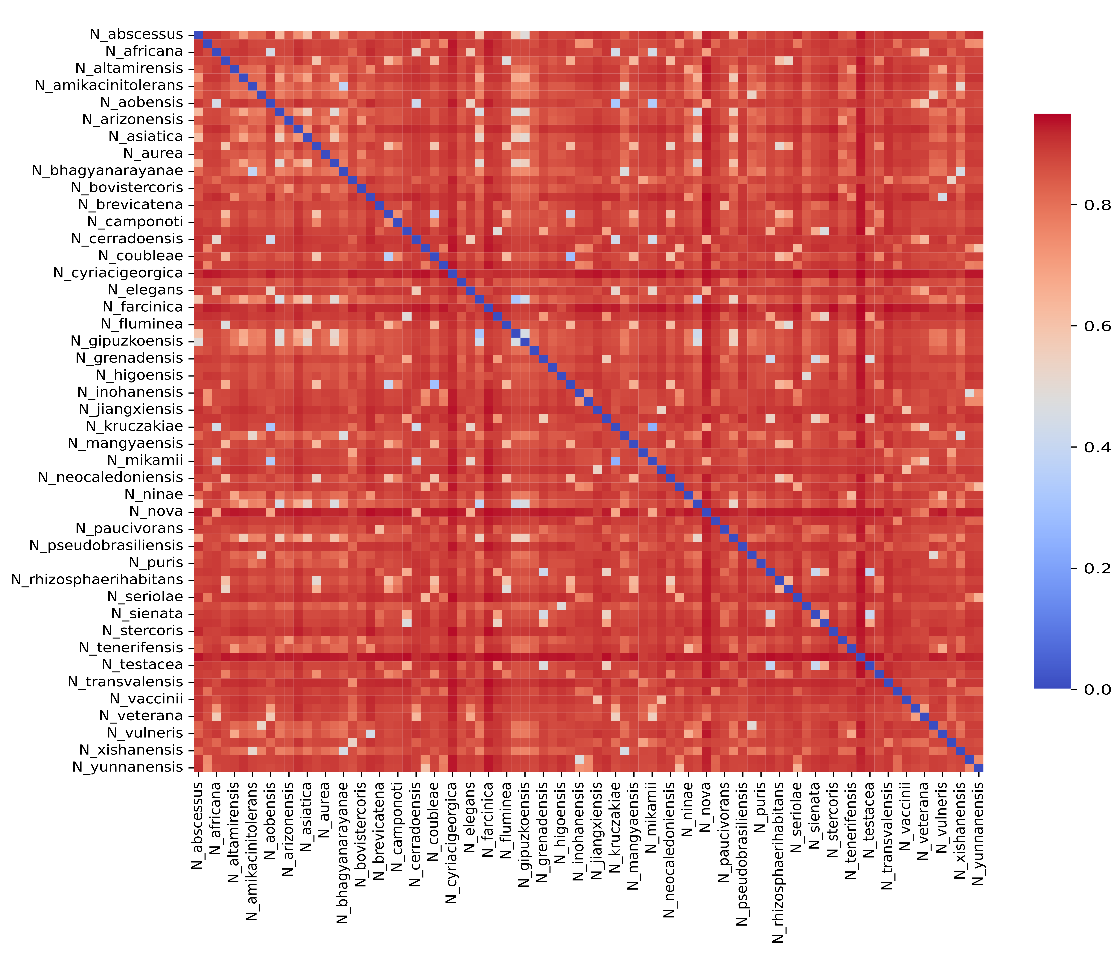


**FIGURE S4.** Pairwise Jaccard distance heatmap of *Nocardia* species. The color gradient represents the Jaccard distance, with darker red shades indicating higher gene content dissimilarity and lighter shades (towards blue) representing greater similarity.


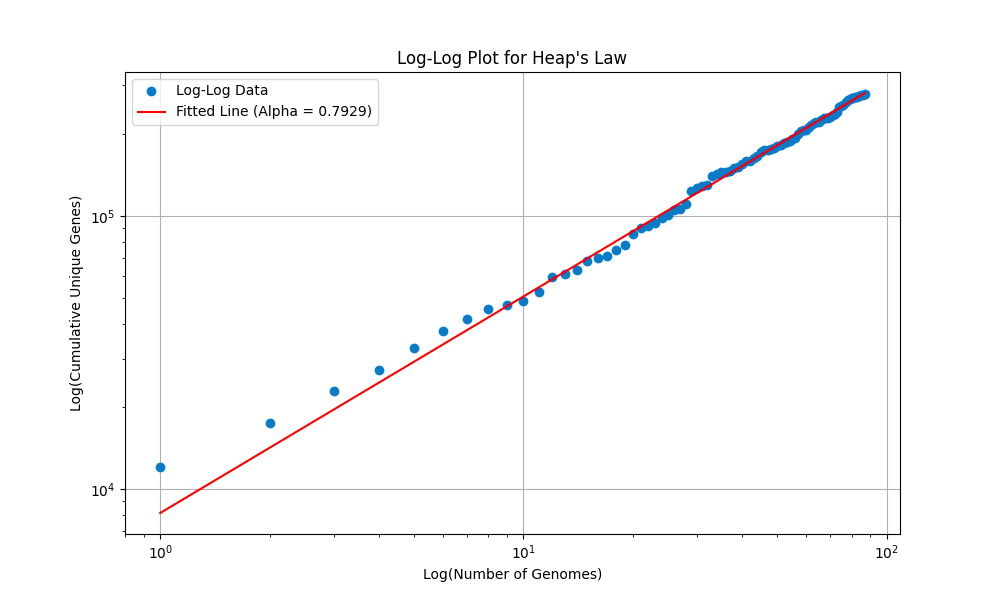


**FIGURE S5.** Log-log plot for Heap's Law in *Nocardia* pangenome analysis. This plot shows the relationship between the number of genomes studied (x-axis) and the cumulative number of unique genes (y-axis) in a log-log scale, following Heap's Law. The log-log plot shows the accumulation of unique genes with increasing *Nocardia* genomes. Blue dots represent observed data and the red fitted line (α = 0.80) indicates an open pangenome (i.e., new genes are discovered as more genomes are added).


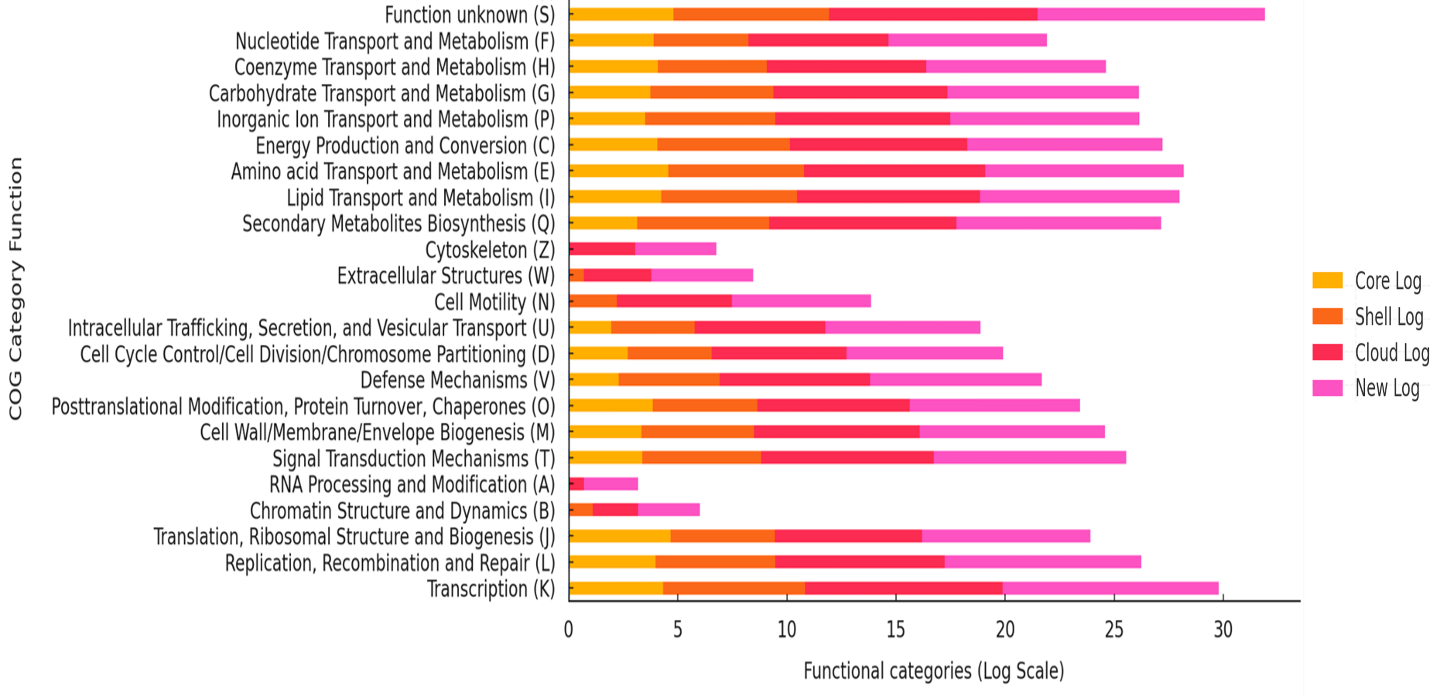


**FIGURE S6.** COG category distribution across core, shell, cloud, and new genes. This figure shows the log10-transformed distribution of core genome, shell genome, cloud genome, and new genes across COG categories in the 87 *Nocardia* species studied.


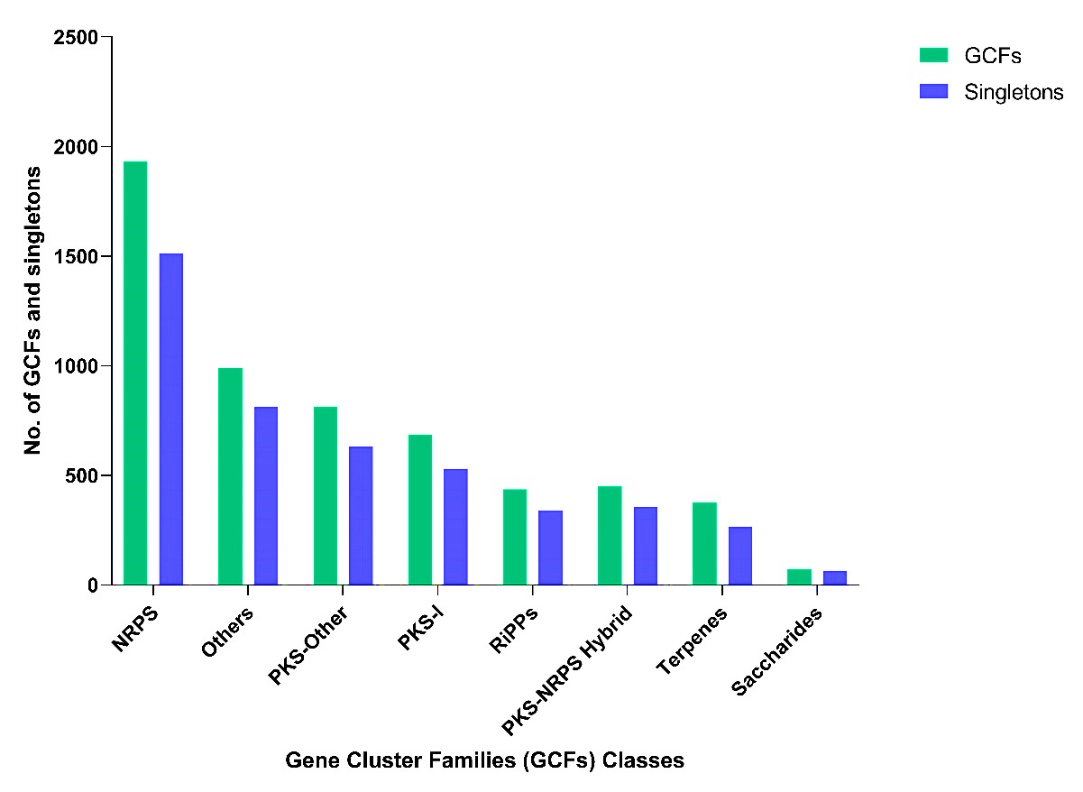


**FIGURE S7.** Distributions of gene cluster families (GCFs) and singletons. The X-axis shows different GCF categories, while the Y-axis represents the counts of GCFs and singletons.

**
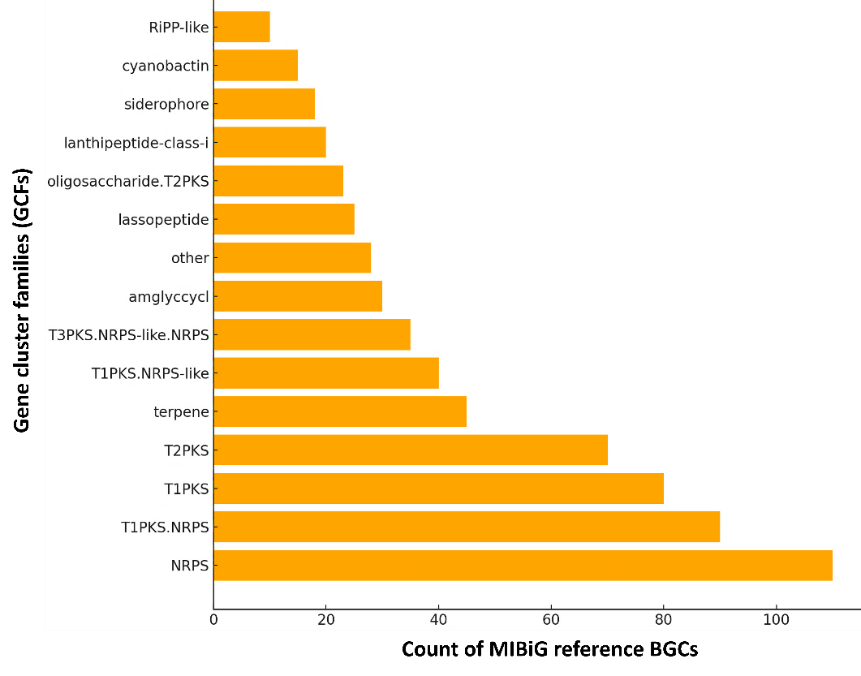
**

**FIGURE S8.** MIBiG reference BGC counts across GCFs. The distribution of reference BGC counts from MIBiG in the top 15 gene cluster families (GCFs) of *Nocardia* genomes.
